# Knowledge and attitude of Malaysian university students towards regenerative medicine therapy and their clinical implications: a cross-sectional study

**DOI:** 10.3389/fgene.2026.1804348

**Published:** 2026-07-28

**Authors:** Narmadaa Raman, Fazlina Nordin, Siti A. M. Imran, Budi Aslinie Md Sabri, Deny Susanti, Khairul Bariah Ahmad Amin Noordin, Sharifah Zamiah Syed Abdul Kadir, Rajesh Ramasamy

**Affiliations:** 1 Department of Tissue Engineering and Regenerative Medicine (DTERM), Faculty of Medicine, Universiti Kebangsaan Malaysia (UKM), Kuala Lumpur, Malaysia; 2 Ming Medical Sdn. Bhd., Petaling Jaya, Selangor, Malaysia; 3 Faculty of Dentistry, Universiti Teknologi Mara (UiTM), Shah Alam, Selangor, Malaysia; 4 Department of Chemistry, Kuliyyah of Science, International Islamic University Malaysia (IIUM), Kuantan, Pahang, Malaysia; 5 School of Dental Sciences, Universiti Sains Malaysia (USM), Kubang Kerian, Kelantan, Malaysia; 6 Department of Pharmacology, Faculty of Medicine, Universiti Malaya (UM), Kuala Lumpur, Malaysia; 7 Department of Pathology, Faculty of Medicine and Health Sciences, Universiti Putra Malaysia (UPM), Serdang, Selangor, Malaysia

**Keywords:** attitude, clinical implications, knowledge, Malaysian university students, regenerative medicine theraphy, stem cells

## Abstract

Regenerative medicine therapy (RMT) is an emerging field in Malaysia with significant potential for tissue regeneration and disease treatment. Yet awareness of its importance and the benefits of stem cell therapy remains limited, owing to a lack of in-depth knowledge of RMT among Malaysians. This study aims to evaluate the knowledge and attitudes of students at public and private universities in Malaysia regarding regenerative medicine therapy and its clinical implications. A cross-sectional study was conducted among 480 medical and non-medical students from Malaysian public and private universities. Six content experts validated the questionnaire, and the appropriateness of each question was rated using an item content validity index (I-CVI). The survey was divided into distinct sections covering demographics, previous experience with RMT, knowledge of RMT development and therapeutic applications (scored binary as 0 or 1), and attitudes towards RMT research, ethics, and religious views (measured on a 5-point Likert scale). The knowledge section categorised levels as poor (0–4), moderate (5–8), or high (9–12). Findings revealed that fundamental knowledge of RMT is inadequate among university students, with low overall mean knowledge scores of 0.56 ± 0.23 and 0.47 ± 0.22. Since most students recognised basic concepts, 80.6% failed to correctly identify specific stem cell types, such as chondrocytes, or the source of embryonic stem cells. Medical students demonstrated significantly greater knowledge and understanding of RMT than non-medical students (p < 0.05), particularly in identifying RMT as a treatment for cancer and tissue replacement. These results underscore an urgent need for curriculum reform to prevent future professional negligence and ensure robust healthcare risk management. Students showed a generally positive attitude towards stem cells and their therapeutic applications, with mean scores of 3.75 ± 0.53 and 3.65 ± 0.56, respectively, showing no significant difference between females and males (p > 0.05). In conclusion, Malaysian university students have a moderate to positive attitude towards stem cells and their therapeutic applications in regenerative medicine; however, their knowledge is inadequate, suggesting the need to implement a regenerative medicine syllabus in the current curriculum to educate them on required knowledge of stem cells and prepare future healthcare professionals and graduates.

## 1 Introduction

Regenerative medicine holds promise as a therapeutic option for replacing lost or damaged organs or tissues, among the multitude of scientific and biomedical advances (Rasekh and Arshad, 2025). A fundamental paradigm of regenerative medicine is the delivery of therapeutic cells that directly contribute to the growth and development of new tissues (Lyu and Zhang, 2026). This technology can either repair or replace damaged tissues (Raman et al., 2022a). Thus, cells utilised in these therapeutic approaches are generally differentiated, allogeneic, or autologous and retain their proliferative capacity (Altyar et al., 2023). Through years of investigation and analysis, valuable knowledge has been gained regarding this extremely essential yet intricate survival mechanism. TH Morgan, a geneticist and evolutionary biologist from the United States, categorised regeneration mechanisms according to the existence or absence of cellular proliferation by leveraging these regeneratively produced tissues, ultimately leading to the investigation of disease mechanisms, diagnostic procedures, and potential treatments (Naranjo et al., 2016). Furthermore, they are employed in fabricating extracorporeal life support systems and for the rehabilitation of injuries or organ replacement via implantation (Kou et al., 2024). Numerous regenerative medicine therapies, including those developed for orthopaedics and wound healing, have been granted commercial availability by the Food and Drug Administration (FDA) in addition to the clearance for human trials of encapsulated stem cell-derived pancreatic islets (NCT05791201) (Naranjo et al., 2016). Despite these clinical breakthroughs, the translation of regenerative strategies into broader medical practice remains challenged by complexities such as organ scarcity, immune rejection, and the need for sophisticated tissue mimics (Bhutambare et al., 2024). Addressing these limitations requires an integrated approach that combines advancements in biomimetic construct fabrication, materials science, and bioengineering to replicate physiological environments (Addissouky, 2025; Dixit et al., 2025). Furthermore, identifying and overcoming existing hurdles within the field is essential to ensure that patient-specific therapies become a consistent and efficacious clinical reality (Xiong et al., 2022; Li, 2026).

Mesenchymal stem cells hold great promise, as their ability to self-renew and differentiate into various cell types presents exceptional potential for advancements in RMT applications (Raman et al., 2022b). Beyond their differentiation potential, these cells exert significant immunomodulatory effects and facilitate tissue repair through paracrine signalling, serving as critical components in modern regenerative strategies (Fonteles et al., 2024; Sajjad et al., 2024). The emergence of organoid technology and microfluidic systems further exemplifies this evolution, allowing for the precise control of biochemical microenvironments and enhanced structural maturation of engineered tissues (Akhtar and Gupta, 2024; Kim and Lee, 2025; Trigo et al., 2025). However, translating these complex tissue-engineered products into widespread clinical use remains dependent on identifying optimal, non-immunogenic cell sources and refining scaffold architectures to ensure long-term functionality (Chen and Liu, 2017). Moreover, addressing scalability and manufacturing consistency is imperative to mitigate risks associated with potential foreign body reactions and to facilitate the regulatory approval of such advanced therapies (Barbon et al., 2025). Achieving these milestones will require rigorous monitoring of stem cell isolation and their microenvironments to enhance both the safety profiles and the overall therapeutic efficacy of these interventions (Kalodimou, 2024).

In the realm of regenerative medicine, biomaterials are indispensable, notably in the restoration of injured tissues and organs, alongside addressing chronic diseases to reinstate regular bodily function (Sanchez Armengol et al., 2024). By mimicking native cellular microenvironments, these therapies aim to transcend current limitations, thereby driving advancements that possess the potential to transform standard healthcare outcomes (Bin Akhtar and Das Gupta, 2024). Ensuring ideal conditions for cell growth remains a primary hurdle in using biological products in cell therapy (Sampogna et al., 2015). Hence, biomaterials are employed to address these obstacles via regulating cellular responses, encompassing interactions between cells and between cells and the extracellular matrix (Dzobo et al., 2018; Hoang, 2025). Additionally, they serve as scaffolds, furnishing structural support for cell adhesion and tissue maturation (Fauzi et al., 2016). Tissue engineering comprises three essential elements, which are reparative cells capable of generating functional matrices, suitable scaffolds for transplantation, and bioactive molecules that stimulate and orchestrate the creation of the targeted tissue (Lightner and Chan, 2021; Liu et al., 2022) components can be exploited individually or in conjunction to facilitate the regeneration of organs or tissue (Meiyappan et al., 2020; Dutta et al., 2025).

The greatest benefits have emerged from stem cell therapies such as regenerative therapies harnessing adult stem cells, which have proven effective in treating hematologic malignancies, burn injuries, and ocular degeneration (McKinley et al., 2023; Wei et al., 2024). These treatment methods are also adaptable for genetic editing to address single-gene abnormalities (El Nahas et al., 2024). Human pluripotent stem cell (hPSC) based therapies have shown potential and are currently in clinical trials in the United States for type 1 diabetes (T1D; clinical trial NCT04786262), Parkinson’s disease (PD; NCT02452723 and NCT03119636), and age-related macular degeneration (AMD; NCT04339764) (Calafiore et al., 2024). These conditions are particularly suited for stem cell-based treatments due to their association with the deficiency of specific cell types: insulin-producing pancreatic islets in T1D, midbrain dopaminergic (mDA) neurons in PD, and retinal pigment epithelial cells in AMD (Hogrebe et al., 2023). Additionally, the transplantation sites are easily accessible via surgery (Ghoneim et al., 2024). Laboratory-generated hPSCs are currently being evaluated for safety and effectiveness in mitigating disease symptoms.

In Malaysia, mesenchymal stem cells (MSCs) and hematopoietic stem cells (HSCs) are the most commonly used stem cells in Regenerative Medicine Therapy (RMT). Hospital Ampang Clinical Stem Cell Services is recognised as one of the top hospitals for stem cell therapy, with a particular focus on blood cancer treatment, such as leukaemia (Imran et al., 2022). MSCs are frequently employed in regenerative medicine for bone regeneration and are being investigated for their potential in treating various malignancies. Platelet-rich plasma (PRP) treatment is less common but practised, particularly at Hospital Universiti Sains Malaysia (HUSM), mainly for treating osteoarthritis (Blaga et al., 2024). Hospital Ampang, prominent as the National Referral Centre for Haematological Expertise, has significantly increased its cell therapy transplant procedures to around 180 per year, with a reduced waiting period of less than 2 months compared to the initial 6 months (Rahman et al., 2022). The Malaysian Stem Cell Therapy Working Group (SCM) comprises medical professionals and specialists offering advanced regenerative medicine services across countries, including at partner clinics in the Klang Valley (the Malaysian Medical Council, 2009). Although data on treatments performed at these partner clinics are unavailable, HUSM specialists have been practising PRP treatment since 2018, focusing on skin rejuvenation, wrinkle reduction, and addressing abnormalities caused by acne marks (Banihashemi et al., 2021; Imran et al., 2022; Phoebe et al., 2024).

In the pursuit of regenerative therapies, the 6th Asia Partnership Conference of Regenerative Medicine (APACRM) took place online on 20 April 2023, aiming to promote regulatory alignment for regenerative medicine products across Asia. Acknowledging the diversity of domestic regulatory guidelines within each country and region is crucial as an initial step toward regulatory convergence (Karasawa et al., 2024). The conference was focused on evaluating non-clinical, quality, and environmental impact assessment settings for cell and gene therapy products. Industry presentations and panel discussions with regulatory agencies highlighted challenges in coordinating regulations, particularly regarding environmental impact assessment (EIA) reviews, due to country-specific reasons and situations (Mahmud, 2022; Karasawa et al., 2024). Despite these challenges, the conference emphasised the importance of seeking more streamlined and effective EIA evaluations by considering the perspectives of each country and region (George et al., 2020). Regulatory oversight is crucial to safeguarding biotechnology-derived cells and tissues as therapeutic products. Global drug regulatory agencies, such as those in the United States, Canada, the European Union, Singapore, Japan, and Korea, recognised cell therapy products as drugs or medicinal products (Yoon et al., 2024). However, these agencies acknowledge that the current drug regulatory framework may not be the most suitable for regulating these products, leading to the establishment or development of separate frameworks specifically for cell therapy products (Mohd Halimi et al., 2025).

Malaysia currently lacks comprehensive legislation dedicated to Regenerative Medicine Therapies for either clinical practice or research. At the same time, this global complexity is mirrored by a fragmented, guideline-based oversight, leaving many advanced applications categorised as experimental. The existing regulations, outlined in 2009 guidelines, mainly permit hematopoietic stem cell and umbilical cord stem cell transplantations, while other types of stem cells, such as mesenchymal stem cells and pluripotent stem cells, are considered experimental (the Malaysian Medical Council, 2009; Mohd Halimi et al., 2025). Xenotransplantation and therapies that involve animal stem cells or animal cells are prohibited. Private healthcare facilities intending to perform must be licensed under the Private Healthcare Facility and Services Act 1998, with guidelines primarily based on those used by the US FDA. There is a call for specific legislation in Malaysia on RMT application for aesthetic purposes and disease treatments. Such legislation would help regulate and monitor the proper application of RMT in the country, preventing misuse or harmful advertising (Hawthorne, 2024). Additionally, guidelines for cell and gene therapy products (CGTPs) were introduced in 2021 by the National Pharmaceutical Regulatory Agency (NPRA), focusing mainly on compliance with good manufacturing practices (GMP) (Mohd Halimi et al., 2025). However, more detailed and focused guidelines were deemed necessary to ensure higher standards in producing CGTPs, particularly due to their involvement in treating severe and chronic illnesses, besides handling live cells (Hawthorne, 2024). This legislative vacuum makes it vital for students to attain high levels of regulatory literacy; they must be educated to navigate these domestic “patchwork” regulations and distinguish between validated, guideline-compliant clinical treatments and the misleading commercial claims or harmful advertising that often target the public (Reinders Folmer et al., 2026).

Medical and allied medical students are expected to have a strong understanding of stem cells and a positive attitude toward their medical applications. However, worldwide studies indicate significant variation in students’ knowledge and attitudes toward stem cells and regenerative medicine. For instance, a study by (Al-Shammary and Hassan, 2023) found that nursing students exhibited moderate knowledge levels and a positive attitude toward stem cell therapy. Conversely, a study in Tabuk, Saudi Arabia (ALtemani et al., 2018), revealed suboptimal knowledge, attitude, and practice among final-year medical students and doctors regarding stem cell use in managing diabetes. Education interventions have been shown to improve knowledge and attitudes toward stem cell therapy (Al-shammary and Hassan, 2023). For example, a study in Saudi Arabia involving nursing students found poor knowledge but a positive attitude before an educational intervention. Following the intervention, there was a significant increase in knowledge levels (Abdulrazeq et al., 2022). However, studies indicate inadequate knowledge and awareness among the public and healthcare staff regarding cord blood banking (Peberdy et al., 2016).

In addition to harvesting stem cells for regenerative therapies, scientists have the potential to make significant contributions to the treatment of dental diseases (Dipalma et al., 2025). Previous studies of this can be seen in the potential applications of dental stem cells, which have been documented to repair cranial defects, regenerate neural tissue, and treat cardiac diseases (Omerkić and Omerkić Dautović, 2025). To promote the adoption of stem cells in dentistry and medicine, scientists and dentists should have the necessary understanding of stem cells and maintain a positive perspective on their potential applications. As evidenced by prior research, health professionals and students in health science programs in numerous nations possess differing degrees of understanding and concern regarding stem cells and their potential applications (Almaeen et al., 2021). In Italy, for instance, there was a positive opinion towards the use of stem cells in medicine while complying with law and order; however, their knowledge was inadequate (Frati et al., 2014). Nursing students in Malaysia, alongside students from other health disciplines, exhibited a moderate level of understanding and an optimistic disposition regarding the therapeutic capabilities of stem cells (Lye et al., 2015). A recent study conducted in Saudi Arabia revealed that a specific group of nursing students exhibited an inadequate understanding of stem cells, despite sustaining a good perception of stem cell therapy (Atef, 2024).

The persistent knowledge gaps among Malaysian university students, who display a moderate understanding despite positive attitudes toward regenerative medicine, underscore a critical need for curriculum reform. This necessity is further validated by a study conducted focusing on active medical professionals in Saudi Arabia, which reveals that even experienced practitioners exhibit significant knowledge deficits, particularly concerning specialised resources, such as the Saudi Stem Cell Donor Registry (Alhadlaq et al., 2019). The study found that while clinicians and pharmacists generally outperformed nurses in knowledge, specific subgroups, including female professionals, Saudi nationals, and those lacking prior work experience, demonstrated notably lower levels of awareness and sensitivity regarding the social and ethical complexities of stem-cell therapy (Al-Shammary and Hassan, 2023). These findings provide a more relevant rationale for assessing the next-generation of professionals, as the identified gaps in the current workforce suggest that existing educational frameworks are insufficient to prevent professional negligence and ensure robust healthcare risk management (Ajlan and Ashri, 2025). Consequently, integrating comprehensive regenerative medicine training at the undergraduate level is vital to equip future graduates with the scientific literacy and ethical competency required to lead and safely implement transformative biomedical innovations in clinical practice (Shokoohmand et al., 2026).

Despite the growing clinical footprint of regenerative medicine therapy (RMT) in Malaysia, there remains a critical void in the evidence regarding the readiness of the future healthcare workforce (Phang and Bin Abdul Aziz, 2025). While localised studies have examined specific groups among nursing students, there has not yet been a comprehensive, multi-institutional analysis comparing the scientific literacy of medical and non-medical students or professionals across the public and private sectors (Shokoohmand et al., 2026). This distinction is vital, as the development of Malaysia’s RMT infrastructure relies on collaboration between clinicians and non-medical professionals (Ruan et al., 2025). To the best of our knowledge, there is a lack of published reports assessing Malaysians’ understanding and perspective regarding regenerative medicine and its clinical implications. An evaluation of such interests, especially among university students who represent future professionals, could potentially reveal the extent of their familiarity with regenerative medicine and its effects. This could also help identify knowledge gaps in higher education and convey accurate information about regenerative medicine. Therefore, this paper aims to evaluate the knowledge and attitudes of Malaysian university students toward RMT and its clinical implications. The specific objectives of this research are to identify specific knowledge deficiencies and misconceptions regarding stem cell types and therapeutic applications among medical and non-medical students attending the public and private universities in Malaysia. Furthermore, the present study assesses attitudinal trends toward RMT research, funding, and ethics to determine student readiness for future clinical practice. It also provides data-driven recommendations for integrating a specialised regenerative medicine syllabus into the national higher education curriculum to bridge identified gaps and ensure a future-ready workforce.

## 2 Materials and methods

### 2.1 Study design

**FIGURE 1:**
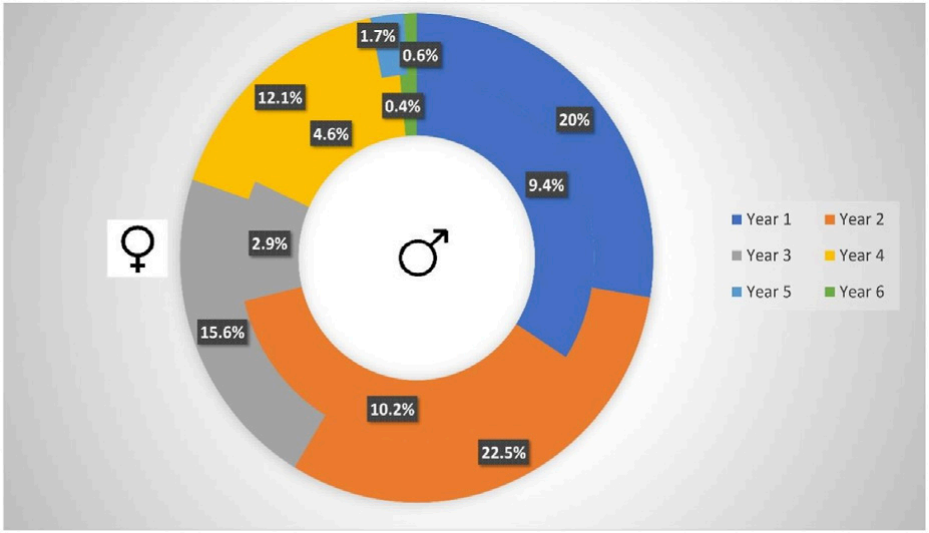
Year of study by gender distribution.

This study is a questionnaire-based cross-sectional study using convenient sampling. An online questionnaire via Google Forms was distributed to undergraduates and postgraduate students at public and private universities. A Google Form was sent to university lecturers to be disseminated to their students during their classes, seminars, and courses (Supplementary 1) from October 2022 until April 2024 (18 months). The link will also be distributed to the public via email and social media applications (Facebook, WhatsApp, and Twitter) (Supplementary 2). The study obtained ethical approval from the Universiti Kebangsaan Malaysia Ethics Committee (Ref: UKM PPI/111/8/JEP-2021- 637). Figure 1 shows a schematic workflow of the proposed RMT survey study.

### 2.2 Questionnaire

The questionnaire was prepared in English to ensure a broader spectrum of respondents and to include international students attending Malaysian public universities. The questionnaire started with informed consent from the respondent, followed by a collection of some demographic data, including age, gender, and educational background. The questionnaire was divided into students and those who are working. Students would be directed to further education background information, namely, the University or College they are attending and their year of study. It further divides those who are in the medical field and the non-medical field. Those who are in the medical field would have to answer four (4) questions in the syllabus and course assessment section. Afterwards, all respondents will be directed to answer questions about their previous experience with the RMT section to assess their experience and exposure to RMT.

The next section of the questionnaire aims to assess the respondents’ basic understanding of the development of RMT and its therapeutic applications, consisting of six questions. The answers for the knowledge section were divided into True, False, and I do n’t know. Respondents may only select one answer for each statement. The correct answer was given a score of (1), and the wrong answer was given a score of (0). The answer “I do n’t know” was also given a score of (0).

The second part of the questionnaire is to assess the attitude of the respondents towards the application of RMT, its application and research in Malaysia, as well as the religious and ethical views of the respondents towards RMT. The attitude portion of the questionnaire consisted of 12 statements that suggested the degree of agreement or disagreement with each statement using a 5-point Likert scale. The answers were divided into three categories based on the total score of the respondents: Strongly/Disagree (1–2), Neutral (3), and Strongly/Agree (4–5). Respondents were instructed to choose only one answer for each statement. Attitude level was divided into four categories based on the total score of each respondent: poor (10–29), moderate (30–39), good (40–44), and excellent (45–50).

The final part of the section is to further assess the respondents’ knowledge and understanding of the involvement of stem cells as part of RMT. This portion of the questionnaire consisted of 12 statements with three possible responses: “Yes”, “No” or “I don’t know”. The scores were assessed similarly to those of the knowledge section.

### 2.3 Questionnaire validation

The questionnaire validation was performed through an online Google platform. Six (6) content experts have validated the questionnaire. Panel members were asked to rate the instrument items in terms of clarity, relevancy, and simplicity on a 4-point ordinal scale (1 [not relevant], 2 [somewhat relevant], 3 [quite relevant], 4 [highly relevant]). To obtain the content validity index of each item (I-CVIs), the number of those judging the item as relevant, simple, or clear (rating 3 or 4) was divided by the number of content experts. If the I-CVI is higher than 79%, the item will be appropriate. If it is between 70% and 79%, it needs revision. If it is less than 70%, it is eliminated. Overall, all questions were validated as appropriate by the validators with an average I-CVI value of 0.997 (Supplementary 3).

### 2.4 Sample size calculation

The proper sample size was calculated using the following equation to ensure statistical strength and representativeness of the population to achieve statistical significance. The calculation was performed using a Z value of 1.96 corresponding to a 95% confidence level, an assumed population proportion (p) of 0.5, representing the percentage of participants expected to select a particular response when no prior estimate is available, and a confidence interval (c) of 0.05, representing a 5% margin of error. Based on these parameters, the minimum required sample size was estimated to be 384 respondents to ensure adequate statistical precision and representativeness of the target population. A total of 480 participants were ultimately recruited, exceeding the minimum sample size requirement and thereby increasing the robustness and reliability of the study findings.

### 2.5 Statistical analysis

The data will be analysed using the Statistical Package for the Social Sciences (Version 22.0, SPSS, Chicago, IL, United States) and presented in a graph using GraphPad Prism. An independent-samples t-test will be used to analyse differences between two independent groups. Pearson’s correlation was used to assess the association between knowledge and attitude, while the Chi-square test was used to assess the association between the study’s variables. The significance level for all tests was set at a 95% confidence interval. Statistical significance will be set to p < 0.05.

## 3 Results

### 3.1 Sample characteristics

Table 1 shows the number of respondents recruited in the study, comprising 11 public and 2 private higher institutions. A total of 480 students were recruited in the present study. 470 (97.92%) respondents were from polytechnic and public universities, with UKM amongst the highest respondents (61.46%). 10 (2.08%) were from private universities. The student ratio of male to female was approximately 1:3, with 132 males and 348 females. Three research universities were involved in the studies: UKM, UM, and USM.

**TABLE 1:** Sample distribution based on polytechnic, universities, and gender (n = 480).

​	Universities	Male	Female	Total
Public	Universiti Kebangsaan Malaysia (UKM)	83	212	295
​	Universiti Malaya (UM)	4	16	20
​	Universiti Sains Malaysia (USM)	7	14	21
​	Universiti Islam Antarabangsa Malaysia (IIUM)	10	32	42
​	Universiti Teknologi MARA (UiTM)	8	33	41
​	Universiti Sultan Zainal Abidin (UniSZA)	11	26	37
​	Universiti Malaysia Terengganu (UMT)	1	0	1
​	Universiti Teknologi Malaysia (UTM)	0	1	1
​	Politeknik Sultan Salahuddin Abdul Aziz Shah (PSA)	4	6	10
​	Politeknik Seberang Perai (PSP)	1	0	1
​	Politeknik Ungku Omar (PUO)	0	1	1
Total	​	129	341	470
Private	Universiti Tunku Abdul Rahman (UTAR)	3	6	9
​	UOW Malaysia KDU	0	1	1
Total	​	3	7	10
Grand total	​	132	348	480

### 3.2 Demographic profile and field of study

Out of the total, almost half were from the non-medical field, comprising 21.31% males and 78.69% females. While 33.90% of males and 66.10% of females were from the medical field, as shown in Table 2. Most of the students from medical (49.17%) and non-medical (50.83%) courses who participated in this study were categorised in the range of 18–23 years old. Moreover, among the male and female students, female students from the medical and non-medical fields prominently participated in this study. Among these 480 students, males account for approximately 9.4% (year 1), 10.2% (year 2), 2.9% (year 3), 4.6% (year 4), and 0.4% (year 6). Additionally, females represented 20% in year 1, 22.5% (year 2), and 15.6% (year 3), and the fewest respondents were from years 4 (12.1%), 5 (1.7%) and 6 (0.6%), respectively (Figure 1).

**TABLE 2:** Percentage of students (n = 480) based on the course of study, age, and gender.

Course of study			Frequency (%)
Medical	Age	18–23	131 (55.51)
		24–29	54 (22.88)
		30–35	43 (18.22)
		36–41	8 (3.39)
	Gender	Male	80 (33.90)
		Female	156 (66.10)
Non-medical	Age	18–23	217 (88.93)
		24–29	24 (9.84)
		30–35	3 (1.23)
		36–41	0 (0)
	Gender	Male	52 (21.31)
		Female	192 (78.69)

### 3.3 Perceptions of RMT course in the current curriculum syllabus

Medical students are required to answer the syllabus, and the course assessment section is tabulated in Table 3. From the findings, a total of 39% of medical students advocate for the incorporation of a specialised course on Regenerative Medicine Therapy (RMT) into the curriculum syllabus. These statements reflect the students’ perspectives on the inclusion and relevance of RMT in their medical education. Approximately 36.9% of students were willing to attend an advanced training course or seminar focused on RMT if such opportunities were made available. Only 34.2% of the students believe their current curriculum provides adequate information regarding RMT and its potential applications in specific diseases and injuries. Merely 25.8% of students have engaged in scientific activities such as seminars, forums, and non-scientific activities, including public gatherings related to RMT.

**TABLE 3:** Syllabus assessments among the medical students (n = 236).

Syllabus assessments	Strongly disagreed n (%)	Disagreed n (%)	Neutral n (%)	Agreed n (%)	Strongly agreed n (%)	Total n (%)
1. The curriculum that I have studied contained a good amount of information about regenerative medicine therapy (RMT) and its potential applications in certain diseases and injuries	6 (2.6)	16 (6.8)	54 (23.1)	78 (33.3)	80 (34.2)	234 (100)
2. Adding a special course concerning RMT to the curriculum syllabus is advisable	3 (1.3)	7 (3.0)	49 (20.8)	85 (36.0)	92 (39.0)	236 (100)
3. I have attended either scientific activities (seminar/forum/courses) or non-scientific activities (public gathering) related to RMT	39 (16.5)	47 (19.9)	42 (17.8)	47 (19.9)	61 (25.8)	236 (100)
4. I Am interested in attending an advanced training course or seminar about RMT if available	2 (0.8)	8 (3.4)	47 (19.9)	92 (39.0)	87 (36.9)	236 (100)

### 3.4 Experience and exposure with RMT

**FIGURE 2:**
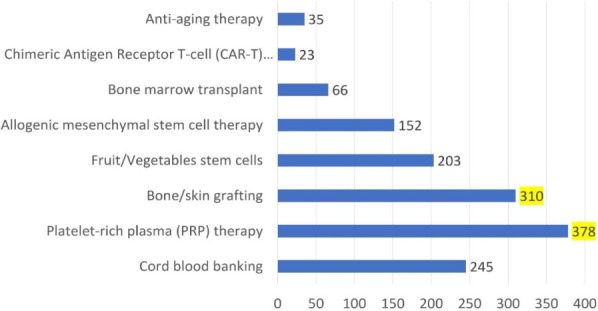
Frequency of correctness of previous experience in RMT.

To assess students' experience and exposure to RMT from their previous experience in the RMT section (Figure 2), they were directed to the questionnaire with eight choices of answers. In this section, students were allowed to choose multiple answers if they felt those were correct statements. The finding showed 378 of 480 (78.75%) students were exposed to platelet-rich plasma (PRP) therapy, followed by 310 (64.58%) students who answered to bone/skin grafting therapy, 152 (31.67%) allogeneic mesenchymal stem cell therapy, 66 (13.75%) bone marrow transplant, and 35 (7.29%) students opted for anti-ageing therapy. These findings suggest that most students had fairly good knowledge based on their previous experience with RMT. Interestingly, a total of 245 (51.04%) of these students selected cord blood, 203 (42.29%) voted for fruit/vegetable stem cells, and 152 (31.67%) opted for chimeric antigen receptor T-cell (CAR-T) therapy, indicating the inaccurate answers. The result revealed that although with some basic knowledge and exposure towards RMT, due to misconceptions or lack of guidance from an expert in the RMT, some of these students could have been confused. In general, the public always thought that cord blood banking, fruit stem cells in cosmetics or supplements, including CAR-T therapy, are always kind of related to RMT. Thus, it is crucial to educate them on this subject matter.

### 3.5 Knowledge and understanding of RMT towards attitude and practices

The next section aims to assess the students' knowledge and understanding of RMT towards their attitude and practices (Table 4). Most medical students 52.2% know that stem cell therapy uses stem cells to treat or prevent a disease or condition, as compared to 47.8% of non-medical students who answered correctly. However, only 53.6% of medical students answered correctly when asked whether RMT is a treatment for certain diseases, such as cancer-related diseases, injuries, and tissue/organ replacement, and 54.1% of them answered correctly when asked whether the biomaterial was a substance that has been engineered to interact with biological systems for a medical purpose, respectively. Moreover, 54.2% of the medical students answered correctly that RMT involves three main niches of research, which are stem cell therapy, tissue engineering, and biomaterials. The details of the responses of the medical and non-medical field students to the basic understanding questionnaire are shown in Table 4. From the data obtained, medical students were equipped with better knowledge and understanding of RMT compared to non-medical students. It can be concluded that stem cell therapy was the most prominent and common knowledge among medical and non-medical students.

**TABLE 4:** Basic understanding among medical and non-medical students (n = 480).

Basic understanding statements		Course of study				
		Medical		Non-medical		Total
		Frequency	(%)	Frequency	(%)	n (%)
RMT involves three (3) main niches of research which are i) stem cell therapy, ii) tissue engineering, and iii) biomaterials	Incorrect	48	(36.1)	85	(63.9)	133 (100.0)
	Correct	188	(54.2)	159	(45.8)	347 (100.0)
Stem cell therapy is the use of stem cells to treat or prevent a disease or condition	Incorrect	24	(32.4)	50	(67.6)	74 (100.0)
	Correct	212	(52.2)	194	(47.8)	406 (100.0)
Tissue engineering uses cells, materials and engineering to treat diseases	Incorrect	48	(37.8)	81	(62.8)	129 (100.0)
	Correct	188	(53.6)	163	(46.4)	351 (100.0)
A biomaterial is a substance that has been engineered to interact with biological systems for a medical purpose	Incorrect	43	(35.0)	80	(65.0)	123 (100.0)
	Correct	193	(54.1)	164	(45.9)	357 (100.0)
RMT is a treatment of certain diseases (such as cancer related disease) and injuries (cells/tissues/organs related injuries and replacement)	Incorrect	27	(30.0)	63	(70.0)	90 (100.0)
	Correct	209	(53.6)	181	(46.4)	390 (100.0)
There is no specific legislation on the conservation and use of stem cells in RMT?	Incorrect	178	(48.0)	193	(52.0)	371 (100.0)
	Correct	58	(53.2)	51	(46.8)	109 (100.0)
Grand total	​	236	(49.2)	244	(50.8)	480 (100.0)

### 3.6 A basic understanding of stem cells was common among students

Analysis of the individual questions revealed a lack of knowledge regarding Q4: “Embryonic stem cells can be obtained from the umbilical cord” and Q8: “Chondrocytes are considered as one type of stem cells”. A total of 387 (80.6%) students answered Q4 and Q8 incorrectly. In comparison, the highest percentage of correct responses was 372 (77.5%) for Q1 “Stem cells are an unspecialised type of cells which are capable of forming any cell type”, followed by 338 (70.4%) for Q12 “The embryonic stem cells are capable of differentiating into the three germ layers i) ectoderm, ii) mesoderm, and iii) endoderm”. An independent t-test showed a significant difference between the numbers of correct and incorrect answers for all 12 knowledge statements tested in the questionnaires (p < 0.01). Table 5 shows the total number of students and their proportions for each category of knowledge level.

**TABLE 5:** Number and percentage of polytechnic, public and private university students on correct/incorrect responses to knowledge statements (n = 480).

Knowledge statements	No. of correct responses n (%)	No. of incorrect responses n (%)	Total n (%)	*p*-value
1. Stem cells are unspecialised type of cells which are capable of forming any cell type	372 (77.5)	108 (22.5)	480 (100)	*<0.01*
2. Human sperms and eggs are considered a source of adult stem cells	168 (35)	312 (65)	480 (100)	
3. Stem cells obtained from adults are specialised cells that can form either bone or cartilage only	204 (42.5)	276 (57.5)	480 (100)	
4. Embryonic stem cells can be obtained from umbilical cord	93 (19.4)	387 (80.6)	480 (100)	
5. Stem cells obtained either from tissues or organs (such as liver, pancreas, skin, dental, etc.) are considered as adult stem cells	292 (60.8)	188 (39.2)	480 (100)	
6. Adult bone marrow stem cells are usually taken from the spine	259 (54)	221 (46)	480 (100)	
7. Autologous transplant of adult stem cells can fail mainly because of immunogenic reaction	241 (50.2)	239 (49.8)	480 (100)	
8. Chondrocytes are considered as one type of stem cells	93 (19.4)	387 (80.6)	480 (100)	
9. Dental implants derived from stem cells are now available to replace missing teeth	225 (46.9)	255 (53.1)	480 (100)	
10. Stem cell banks are now available in Malaysia	258 (53.8)	222 (46.2)	480 (100)	
11. Three (3) types of stem cells are i) adult stem cells, ii) embryonic(or pluripotent) stem cell, and iii) induced pluripotent stem cell (iPSC)	314 (65.4)	166 (34.6)	480 (100)	
12. The embryonic stem cells are capable to differentiate into the three germs layers i) ectoderm, ii) mesoderm, and iii) endoderm	338 (70.4)	142 (29.6)	480 (100)	

### 3.7 Female students showed higher knowledge levels as compared to male students

**FIGURE 3:**
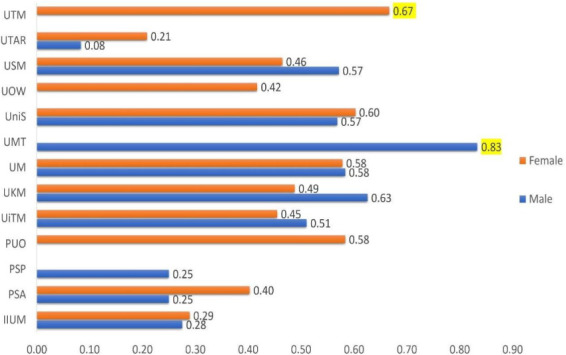
Mean (±SD) knowledge score for males and females.

To facilitate any future study, all information on the knowledge score for all genders was separated. Male students of Universiti Sains Malaysia (USM) showed the highest percentage of moderate knowledge level with 85.7% of correct answers, followed by Universiti Sultan Zainal Abidin (UniSZA) showing 81.8% accordingly. In contrast, students attending Universiti Tunku Abdul Rahman (UTAR) recorded a poorer knowledge level with 100% wrong answers (Table 6). For female students, Universiti Sains Malaysia (USM) showed the highest percentage of moderate knowledge level with 92.9% of correct answers. On the other hand, female students from UTAR garnered a poor knowledge level of 66.7%, with wrong answers, compared to male students. Table 6 shows the frequency and percentage of students in each category of knowledge level based on gender and university. In addition, the analysis showed that there was a significant difference between male students from government institutions with a moderate knowledge level as compared with poor and high knowledge levels (p < 0.0001) (Table 6). A similar pattern was observed among female students from government institutions. The male students from Universiti Malaysia Terengganu (UMT) showed a higher mean knowledge score of 0.83, followed by the female students' mean score of 0.67 in Universiti Teknologi Malaysia (UTM). Overall, female students achieved higher mean knowledge scores compared to male students. Figure 3 shows the mean knowledge scores for males and females from public and private universities.

**TABLE 6:** Number and percentage of students in each category of knowledge level based on gender and university (n=480).

Gender	​	Polytechnic and university	Poor n (%)	Moderate n (%)	High n (%)	Total n (%)	*p*-value
Male	Government	1. Universiti Islam Antarabangsa Malaysia (IIUM)	7 (70.0)	3 (30.0)	0 (0.0)	10 (100.0)	​
		2. Universiti Teknologi MARA (UiTM)	2 (25.0)	5 (62.5)	1 (12.5)	8 (100.0)	
		3. Universiti Kebangsaan Malaysia (UKM)	10 (12.0)	37 (44.6)	36 (43.4)	83 (100.0)	
		4. Universiti Malaya (UM)	1 (25.0)	1 (25.0)	2 (50.0)	4 (100.0)	
		5. Universiti Malaysia Terengganu (UMT)	0 (0.0)	0 (0.0)	1 (100.0)	1 (100.0)	
		6. Universiti Sultan Zainal Abidin (UniSZA)	1 (9.1)	9 (81.8)	1 (9.1)	11 (100.0)	
		7. Universiti Sains Malaysia (USM)	0 (0.0)	6 (85.7)	1 (14.3)	7 (100.0)	
		8. Politeknik Sultan Salahuddin Abdul Aziz Shah (PSA)	3 (75.0)	1 (25.0)	0 (0.0)	4 (100.0)	
		9. Politeknik Seberang Perai (PSP)	1 (100)	0 (0.0)	0 (0.0)	1 (100.0)	
		Total	25 (19.4)	62 (48.0)	42 (32.6)	129 (100.0)	*<0.0001*
	Private	1. Universiti Tunku Abdul Rahman (UTAR)	3 (100.0)	0 (0.0)	0 (0.0)	3 (100.0)	​
		Total	3 (100.0)	0 (0.0)	0 (0.0)	3 (100.0)	​
	​	Grand Total	28 (21.2)	62 (47.0)	42 (31.8)	132 (100.0)	​
Female	Government	1. Universiti Islam Antarabangsa Malaysia (IIUM)	21 (65.6)	11 (34.4)	0 (0.0)	32 (100.0)	*<0.0001*
		2. Universiti Teknologi MARA (UiTM)	12 (36.4)	18 (54.5)	3 (9.1)	33 (100.0)	
		3. Universiti Kebangsaan Malaysia (UKM)	67 (31.6)	113 (53.3)	32 (15.1)	212 (100.0)	
		4. Universiti Malaya (UM)	1 (6.3)	12 (75.0)	3 (18.7)	16 (100.0)	
		5. Universiti Sultan Zainal Abidin (UniSZA)	1 (3.8)	20 (77.0)	5 (19.2)	26 (100.0)	
		6. Universit iSains Malaysia (USM)	1 (7.1)	13 (92.9)	0 (0.0)	14 (100.0)	
		7. Universiti Teknologi Malaysia (UTM)	0 (0.0)	1 (100.0)	0 (0.0)	1 (100.0)	
		8. Politeknik Sultan Salahuddin Abdul Aziz Shah (PSA)	2 (33.3)	4 (66.7)	0 (0.0)	6 (100.0)	
		9. Politeknik Ungku Omar (PUO)	0 (0.0)	1 (100.0)	0 (0.0)	1 (100.0)	
		Total	105 (30.8)	193 (56.6)	43 (12.6)	341 (100.0)	
	Private	1. Universiti Tunku Abdul Rahman (UTAR)	4 (66.7)	2 (33.3)	0 (0.0)	6 (100.0)	​
		2. UOW Malaysia KDU	0 (0.0)	1 (100.0)	0 (0.0)	1 (100.0)	​
		Total	4 (57.1)	3 (42.9)	0 (0.0)	7 (100.0)	​
	​	Grand Total	109 (31.3)	196 (56.3)	43 (12.4)	348 (100.0)	​

### 3.8 Most of the students have a positive attitude towards RMT

The number and percentage of responses to attitude statements among polytechnic, public, and private university students are presented in Table 7. In these analyses, the percentages of strongly agree and agreed were added and considered positive responses. A total of 86.2% (35.0% + 51.2%) of students agreed that the government should put more funding into regenerative medicine therapy-related research, while 83.6% (39.3% + 42.3%) suggested that RMT research should be carried out in Malaysia. Among 85.6% (34.6% + 51.0%) of them emphasised that there should be more public awareness programs about regenerative medicine, and their therapeutic application should be made available to all, which reflected those who agreed with the positive future of RMT research 85.2% (33.7% + 51.5%). Interestingly, a high percentage of students, 81.5% (32.5% + 49.0%), showed keenness in developing knowledge about stem cells, as it is crucial for RMT application for future uses (Table 7).

**TABLE 7:** Number and percentage of responses to attitude statements among polytechnic, public, and private university students (n=480).

Attitude statement	Strongly disagreed n (%)	Disagreed n (%)	Neutral n (%)	Agree n (%)	Strongly agreed n (%)	Total n (%)
1. Do you think that you have enough knowledge of RMT research and its potential applications in the treatment of certain diseases and injuries?	103 (21.5)	131 (27.3)	136 (28.3)	75 (15.6)	35 (7.3)	480 (100)
2.There is good amount of information about RMT and their potential applications in certain diseases and injuries made available to public.	33 (8.1)	102 (21.3)	163 (34.0)	114 (23.7)	62 (12.9)	480 (100)
3. Do you agree that RMT research should be carried out in Malaysia?	3 (0.6)	12 (2.5)	64 (13.3)	163 (34.0)	238 (49.6)	480 (100)
4. Do you think that RMT research has a positive future?	2 (0.4)	9 (1.9)	60 (12.5)	162 (33.7)	247 (51.5)	480 (100)
5. The use of treatment related RMT in medical treatments contradicts with human ethics and religious principle.	69 (14.4)	95 (19.8)	185 (38.5)	74 (15.4)	57 (11.9)	480 (100)
6. I will recommend treatment related to RMT if it is available to my family/friends/public.	5 (1.1)	13 (2.7)	124 (25.8)	169 (35.2)	169 (35.2)	480 (100)
7. I am aware of RMT research and its potential applications in treating certain diseases and injuries.	10 (2.0)	31 (6.5)	154 (22.7)	109 (37.3)	179 (31.5)	480 (100)
8. There should be more public awareness programs about RMT, and their therapeutic application should be made available.	2 (0.4)	9 (1.9)	58 (12.1)	166 (34.6)	245 (51.0)	480 (100)
9. Do you think that the government should put more funding in research related to RMT?	2 (0.4)	9 (1.9)	55 (11.5)	168 (35.0)	246 (51.2)	480 (100)
10. Some people believe that life begins at conception, and that to destroy a human embryo would be immoral, even to save or reduce suffering in an existing human life. Do you agree with this statement?	52 (10.8)	90 (18.8)	194 (40.4)	82 (17.1)	62 (12.9)	480 (100)
11. Many religions also oppose stem cell research and treatment. Do you think it’s acceptable for some parents to refuse stem cell treatment for their children as it goes against their religious beliefs, even if the treatment could be lifesaving?	59 (12.2)	90 (18.8)	175 (36.5)	101 (21.0)	55 (11.5)	480 (100)
12. Would you be interested in developing your knowledge about stem cells?	2 (0.4)	9 (1.9)	78 (16.2)	156 (32.5)	235 (49.0)	480 (100)

More than half of the students, 70.4% (35.2% + 35.2%), were willing to recommend treatment related to RMT if it is available to family, friends, and the surrounding community, since plenty of students, 32.5% (21.0% + 11.5%), believed that the use of stem cell research and treatment did not contradict ethical and religious principles (Table 7). Moreover, 68.8% (37.3% + 31.5%) of them know RMT research and its potential applications in treating certain diseases and injuries. However, 22.9% (15.6% + 7.3%) of the students reported that they still lacked knowledge of RMT research and their potential applications in the treatment of certain diseases and injuries, even though 36.6% (23.7% + 12.9%) of students mentioned there is a good amount of information about RMT and their potential applications in certain diseases and injuries made available to the public (Table 7).

**FIGURE 4:**
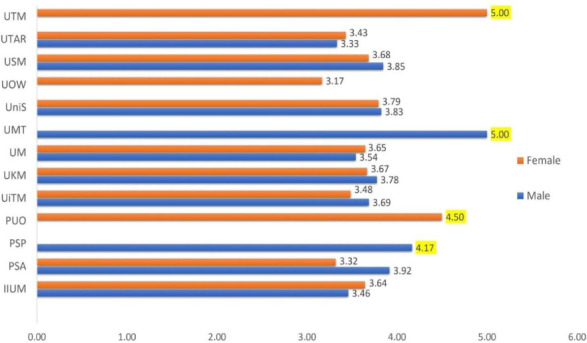
Mean (±SD) attitude score for gender.

In addition, female students from UTM and male students from UMT recorded the highest mean scores, with a mean attitude score of 5.00. Female students from PUO showed a mean attitude score of 4.50, followed by male students in PSP with a mean attitude score of 4.17. Figure 4 shows the mean attitude score for the gender distribution of public and private university students. The attitude levels across polytechnic, government, and private institutions are demonstrated in Table 8, among 66.1% of public and private university students have good attitudes towards RMT application, while only 23.3% of students have an excellent attitude across the study. Overall, 95% of students from UM showed a good attitude towards RMT, followed by 85.7% of students from IIUM. Meanwhile, 60% of private university students demonstrate a moderate attitude towards the applications of RMT. In addition, there was a significant difference between private university students with moderate attitude levels as compared to those with either good or excellent (p < 0.0001). In contrast, the government university students with good attitudes were significantly higher as compared with either poor, moderate or excellent (p < 0.0001).

**TABLE 8:** Attitude level based on university (n=480).

Polytechnic and University	Poor n (%)	Moderate n (%)	Good n (%)	Excellent n (%)	Total n (%)	*p*-value
Government						
1. Universiti Islam Antarabangsa Malaysia (IIUM)	0 (0.0)	2 (4.8)	36 (85.7)	4 (9.5)	42 (100)	​
2. Universiti Teknologi MARA(UiTM)	0 (0.0)	7 (17.1)	29 (70.7)	5 (12.2)	41 (100)	
3. Universiti Kebangsaan Malaysia (UKM)	5 (1.7)	26 (8.8)	187 (63.4)	77 (26.1)	295 (100)	
4. Universiti Malaya (UM)	0 (0.0)	0 (0.0)	19 (95.0)	1 (5.0)	20 (100)	
5. Universiti Malaysia Terengganu (UMT)	0 (0.0)	0 (0.0)	0 (0.0)	1 (100.0)	1 (100)	
6. Universiti Sultan Zainal Abidin (UniSZA)	0 (0.0)	0 (0.0)	27 (73.0)	10 (27.0)	37 (100)	
7. Universiti Sains Malaysia (USM)	0 (0.0)	1 (4.7)	14 (66.7)	6 (28.6)	21(100)	
8. Universiti Teknologi Malaysia (UTM)	0 (0.0)	0 (0.0)	0 (0.0)	1 (100.0)	1 (100)	
9. Politeknik Sultan Salahuddin Abdul Aziz Shah (PSA)	0 (0.0)	4 (40.0)	3 (30.0)	3 (30.0)	10 (100)	
10. Politeknik Seberang Perai (PSP)	0 (0.0)	0 (0.0)	0 (0.0)	1 (100.0)	1 (100)	
11. Politeknik Ungku Omar (PUO)	0 (0.0)	0 (0.0)	0 (0.0)	1 (100.0)	1 (100)	
Total	5 (1.1)	40 (8.5)	315 (67.0)	110 (23.4)	470 (100)	*<0.0001*
Private						
12. Universiti Tunk Abdul Rahman (UTAR)	0 (0.0)	6 (66.7)	1 (11.1)	2 (22.2)	9 (100)	​
13.UOW Malaysia KDU	0 (0.0)	0 (0.0)	1(100)	0 (0.0)	1 (100)	
Total	0 (0.0)	6(60)	2 (20.0)	2 (20.0)	10 (100)	*<0.0001*
Grand total	5 (1.0)	46 (9.6)	317 (66.1)	112 (23.3)	480 (100)	​

### 3.9 Public institution students showed significantly higher knowledge and attitude

The degree of moderate knowledge level shown by students from public institutions was significantly greater than 0.50 ± 0.22 compared to students from private universities, showing 0.19, which indicates a negative correlation (r = −0.463, p < 0.01). Similarly, no statistically significant difference in attitude score was found between government (3.48 ± 0.51) and private institutions (3.27 ± 0.68) (p = 0.19) using an independent sample t-test. The mean attitude score among polytechnic, public, and private university students is shown in Table 9. No significant difference (p = 0.94) was found in the mean knowledge score between male (0.56 ± 0.23) and female (0.47 ± 0.22) students from public and private universities. No significant difference in attitude between males (3.75 ± 0.53) and females (3.65 ± 0.56) using an independent sample t-test (p = 0.75), shown in Table 10. However, the statistical analysis comparing public and private institutions may be subject to bias, as the number of students from private institutions was relatively low compared to students from public institutions.

**TABLE 9:** Mean score of knowledge and attitude in polytechnic, public, and private universities.

​	University	Frequency	**Mean ± SD
Knowledge	Public	470	0.50 ± 0.22
	Private	10	0.19 ± 0.19
Attitude	Public	470	3.48 ± 0.51
	Private	10	3.27 ± 0.68

** Independent t-test and Pearson correlation.

**TABLE 10:** Mean score of knowledge and attitude among male and female respondents.

​	Gender	Frequency	*Mean ± SD
Knowledge	Male	132	0.56 ± 0.23
	Female	348	0.47 ± 0.22
Attitude	Male	132	3.75 ± 0.53
	Female	348	3.65 ± 0.56

* Independent t-test.

## 4 Discussion

Regenerative medicine is an area of medicine that pertains to the reconstruction or replacement of tissues and organs that have been obliterated by age, disease, or trauma, as well as the correction of congenital anomalies. At present, there is promising pre-clinical and clinical data that suggest the potential of regenerative medicine to treat a variety of conditions, including chronic diseases and acute insults, as well as maladies that affect a wide range of organ systems and contexts, including dermal wounds, cardiovascular diseases and traumas, and cancer treatment (Rahimi Darehbagh et al., 2024). Stem cell therapy was highlighted by most of the respondents in the basic understanding section (Table 4) as a vital regenerative medicine therapy, particularly mesenchymal and haematopoietic stem cells, which are crucial in the treatment of chronic diseases, including leukaemia, bone marrow disorders, autoimmune diseases, and urinary issues (Çiçek and Dinç, 2024). This finding was similar to that reported by (Alhadlaq et al., 2019). However, the applications of stem cell research are constrained by ethical concerns regarding embryonic stem cells, tumour development, and rejection, despite the significant progress made in this field. Nevertheless, numerous limitations persist with the potential to yield substantial improvements in the management of various diseases (Park et al., 2024). Therefore, the study found that stem cells were employed to treat cancer-related diseases, tissue repair, and replacement as major applications of stem cell therapy, indicating that medical and non-medical students were aware of its therapeutic potential (Mannan et al., 2025).

Regenerative medicine has increasingly garnered interest in Malaysia over the years. An increasing number of researchers are investigating the potential applications of RMT in Malaysian clinical settings as an alternative to conventional treatments and as a potentially superior treatment option in the future (Li et al., 2024). An increasing number of local researchers and clinicians are actively exploring the applicability of regenerative medicine and therapy (RMT) within Malaysian healthcare settings, as well as a complementary approach to existing treatment modalities and as a promising alternative with the potential to surpass conventional therapeutic outcomes in the future (Guo et al., 2025). These developments not only highlight the country’s growing research capacity but also signal an important shift towards incorporating cutting-edge biomedical innovations into future clinical practice (Leonov et al., 2025). Continued efforts in research, policy development, and public education will be crucial for Malaysia to harness the therapeutic and societal benefits of regenerative medicine. Compared to other developed nations, Malaysia lags significantly in adopting contemporary medical therapies, largely due to limitations in infrastructure, regulatory frameworks, public awareness, and long-term investment in biomedical innovation (Hehsan et al., 2023). Numerous developments have been documented in Malaysia’s research sector regarding RMTs and their clinical implications.

Consequently, medical and non-medical students need to comprehend RMT and its potential applications. This study assessed the level of knowledge and attitudes towards RMT among medical and non-medical students in Malaysian public and private institutions. Students are expected to possess limited knowledge and awareness of regenerative medicine therapy (RMT) due to insufficient exposure to the field, as RMT is still considered a relatively new technology. This step is crucial for examining the current exposure of medical and non-medical students to the concept of RMT. However, the previous study from Meiyappan et al. (2020), depicts that physicians in the 25–35 age group have a significantly higher level of knowledge (p = 0.033). Additionally, speciality (p = 0.00), gender (p = 0.003), and place of graduation (p = 0.009) were significant factors influencing the level of knowledge.

From the findings, it can be concluded that the majority of students were interested in learning more about stem cell research. However, the high failure rate observed in this study, particularly with 80.6% of students incorrectly answering Q4 regarding the source of embryonic stem cells and Q8 regarding the nature of chondrocytes, provides critical insight into the specific nature of the knowledge gap. This data suggests a fundamental confusion between adult stem cells and pluripotent stem cells. While students are likely aware of the clinical relevance of the umbilical cord due to the prevalence of cord blood banking, they fail to distinguish that these are adult (hematopoietic or mesenchymal) sources rather than embryonic ones. Furthermore, the inability to identify chondrocytes as specialised, differentiated cells rather than stem cells points to a significant deficiency in basic histology. This indicates that while students may have a general awareness of RMT applications, such as cartilage repair, they lack the technical understanding of the cellular building blocks required for such therapies (Kausar et al., 2025).

To address these technical misconceptions, it is recommended that a “histology-to-clinical-application bridge” be incorporated into a specialised RMT syllabus, rather than teaching stem cell biology as a separate or abstract concept. The curriculum should explicitly link the biological characteristics of specific cell types to their regulated clinical uses. For example, educational modules should clearly contrast the limited potency of adult stem cells found in the umbilical cord with the pluripotency of embryonic stem cells to correct the identified source-based confusion. By grounding RMT education in a robust histological framework, Malaysian universities can move students beyond moderate general awareness toward the technical literacy necessary to distinguish between validated therapies and experimental research, ultimately preparing a more competent and future-ready workforce. Furthermore, by bridging these technical gaps through targeted educational reforms, universities can empower students to act as informed advocates within the broader healthcare ecosystem, effectively closing the divide between complex biomedical innovation and public understanding (Trinh and Turner, 2025). Equipping future professionals with the capacity to clearly communicate the distinctions between validated clinical applications and experimental research is essential for mitigating the spread of misinformation and fostering realistic patient expectations regarding the potential and limitations of regenerative therapies (Trinh and Turner, 2025).

On the other hand (Ramachandren et al., 2019), reported that medical and health science students possessed a greater level of awareness and knowledge of RMT than engineering and physical science students (p < 0.001). Moreover, a study of knowledge and awareness found that knowledge plays an important role in creating awareness, which in turn contributes to RMT therapies/courses, and that these factors are significantly associated (p < 0.05) (Tan et al., 2016). The educational background of medical students did not influence their understanding of stem cell awareness. Furthermore, the students were aware of stem cells, despite the absence of RMT exposure in their medical curriculum. Public commercial resources and the use of stem cells in medical innovation may have contributed to the maximum level of awareness of their clinical application (Lye et al., 2015). Additionally, diverse educational initiatives concerning stem cells must be tailored to align with the religious, cultural, and social contexts of the community. The implementation of stem cell awareness programs necessitates a thorough consideration of the religious, economic, social, and behavioural determinants present within the population. The Ministry of Health ought to prioritise the advancement of stem cell research by regularly hosting awareness talks, thereby fostering a more favourable perspective on the potential of stem cells in research and therapeutic applications (Ministry of Health, 2020).

While stem-cell applications are well-established in treating hematologic malignancies such as leukemia and bone marrow disorders, recent literature underscores a much broader clinical relevance in the field of oncology. The cancer stem cell (CSC) hypothesis reveals that a subpopulation of tumor cells possesses unique self-renewal and differentiation capacities, serving as the fundamental drivers of tumor initiation, progression, and therapeutic resistance across various malignancies. Recent advancements in redox biology further highlight how redox-sensitive transcription factors, such as NRF2 and HIF-1α, are critical in maintaining CSC homeostasis and plasticity, thereby contributing significantly to chemoresistance and metastasis (Sahoo et al., 2024). Furthermore, the identification of glioma stem cells (GSCs) as the “hidden architects” of aggressive brain tumors similar to glioblastoma multiforme illustrates how these cells fuel tumor recurrence and evade conventional therapies. Incorporating these advanced biological insights into university curricula is essential, as it expands the rationale for stem-cell education beyond the traditional scope of regenerative medicine to include the cutting-edge science of oncology, thereby ensuring that the next-generation of healthcare professionals is equipped to lead and implement transformative biomedical innovations. RMT knowledge is essential for medical students beyond the scope of traditional surgery or regenerative medicine, reinforcing the need for its inclusion in the core curriculum.

Accordingly, the Ministry of Higher Education, in collaboration with the Ministry of Health, should play a scrupulous role in implementing specialised courses on Regenerative Medicine into the curriculum syllabus to create more competent professionals in the future (Abu Bakar et al., 2024). University authorities should acknowledge the significance of stem cells and ensure that all medical and non-medical students acquire a comprehensive understanding and advantageous perspectives on this subject. This knowledge will empower them as future professionals to educate, advocate, and enhance the field of regenerative medicine in its clinical setting. Cater seminars and training courses often bring together industry professionals, instructors, and peers, allowing students to build valuable connections that can lead to mentorship, internships, or job opportunities. The ministry should draft strategies to promote quality practices of regenerative medicine via focused seminars and training.

It has brought to light the difficulties that Malaysia must experience to achieve its goal of joining the ranks of other “rising powers” and “traditional players” in the field of newly developed regenerative medicine and its clinical application. Given the present regulatory patchwork in the regulation of human subject clinical research at the global level, Malaysia has endured obstacles in the process of creating regulatory policies that are fit for its local environment and would be able to encourage scientific and medical advancement (Abdul Aziz et al., 2018). It is also necessary for the plans to be able to safeguard the safety of the general public and preserve the positive reputation of this technology.

The limited resources in Malaysia place the country at a disadvantage when compared to other emerging powers such as India and Singapore, which causes the complexities that surround this region to become even more complicated (Zarzeczny and Caulfield, 2009). The legislation, although not currently being implemented, may act as a strategy due to the low barrier it presents for recruiting scientists and business entities to investigate this subject area. Consequently, a small developing nation with constrained funding, resources, and a varied religious background encounters considerable obstacles in stem cell decision-making and policymaking, as highlighted by the lack of regulatory documentation in developing countries such as Malaysia (Kalidasan and Theva Das, 2021).

Lastly, it is essential that the Ministry of Education, in conjunction with the Ministry of Health and with the assistance of other non-governmental organisations (NGOs), continue and broaden their ongoing endeavours to provide the information, knowledge, and implementation of RMT as a modern therapy, as well as a focused campaign on the clinical implications of regenerative medicine, to the entire community in Malaysia (Pînzariu et al., 2025). Over two-thirds of respondents were familiar with stem cells being utilised by dentists in regenerative dentistry to promote root formation after trauma. Additionally, the finding aligns with a pertinent study conducted on dentists in South Africa. A current study revealed that over 50% of dental interns did not believe that the application of stem cells in dentistry conflicted with ethical and religious principles (Seidel et al., 2025).

Due to globalisation, information technology has widespread globally, enabling students to scrutinise all aspects of the latest information regarding regenerative medicine and medical innovation. Information technology has been enhanced due to better-engineered equipment. However, browsing the right information on the right platform is vital in reaching out the right information about regenerative medicine and its innovation, as knowledge is the light to success (Thacharodi et al., 2024). The Ministry of Higher Learning should promote the websites and correct tools that enrich the latest information regarding regenerative medicine to improve the effectiveness and efficiency of the current curriculum. Comprehensive training seminars, forums, and non-scientific activities, including public gatherings related to RMT, may eventually lead to employment in the future among Malaysian youth. The headhunters are going global in seeking skilled employees; thus, training and seminars opened a new dimension in employment (Jamaludin et al., 2023). Those who are skilled in regenerative medicine will gain an edge over others. Therefore, it is necessary to equip oneself with the right qualifications via actively participating and excelling in advanced training courses or seminars on regenerative medicine.

This contrasts with a prior study that indicated numerous nursing students expressed concerns regarding the ethical implications of stem cell usage. The knowledge and attitudes of recently graduated dentists in Saudi Arabia regarding stem cells and their potential therapeutic applications in dentistry are insufficient (Alhadlaq et al., 2019). Italy determined that two-thirds of the participating students lacked specific knowledge regarding stem cells. In India, the knowledge and attitude of dental professionals regarding dental stem cells revealed that 66% of the dental respondents were unaware of the ethical considerations and guidelines related to DSCs provided by the Indian Council of Medical Research (Katge et al., 2017). A significant portion of dental respondents (83%) expressed interest in attending a workshop, conference, or Continuing Dental Education (CDE) program focused on the applications of stem cells. Overall, the majority of students in this study demonstrated a moderate to positive attitude towards stem cells and their therapeutic applications in regenerative medicine; however, their knowledge was insufficient. The findings indicated that a moderate level of knowledge and attitude scores spur a role in the suboptimal practice level of regenerative medicine as a therapeutic option in Malaysia.

## 5 Conclusion

In conclusion, the present study contributes to an in-depth examination of the knowledge and attitudes towards regenerative medicine and its clinical implications among university students in Malaysia. The findings demonstrate that while these students hold a moderate to positive attitude toward stem cells and their therapeutic applications, their knowledge remains inadequate. These results suggest that gaps in knowledge and attitude contribute to the poor practice of regenerative medicine therapy in Malaysia. To ensure a high standard of understanding regarding regenerative medicine and its clinical implications among the Malaysian population, specific measures must be taken into consideration. Curriculum modifications should emphasise not only conceptual learning but also experiential and interdisciplinary components. Such approaches can motivate students to engage deeply with the principles of regenerative medicine, fostering the curiosity, critical thinking, and research skills necessary for future clinical or scientific practice. As regenerative medicine continues to expand globally, there is an urgent need to prepare the next-generation of Malaysian graduates to participate in, contribute to, and lead developments in this transformative field. Therefore, modifying the current curriculum to include regenerative medicine is recommended to equip university students with the required knowledge and motivate them regarding its therapeutic principles, addressing the significant need for future practice in this area. By strengthening education and awareness from the undergraduate level, Malaysia can build a robust, future-ready workforce that contributes to the nation’s long-term healthcare and technological goals. Therefore, modification in the current curriculum based on regenerative medicine is recommended to equip university students with the required knowledge about stem cells and motivate them about the principles of regenerative medicine therapy, as there is a crying need for future practice on utilising stem cells in regenerative medicine therapy. Furthermore, institutions should establish transdisciplinary co-creation platforms that bring together students from diverse fields such as biotechnology, engineering, and social sciences to collaboratively address the ethical and translational hurdles of regenerative medicine, thereby fostering the fundamental scientific literacy necessary for future practitioners. Addressing this ‘crying need' for specialised education is the essential step toward achieving the nation’s long-term healthcare goals and ensuring the secure, ethical implementation of transformative medicine.

## 6 Strengths and limitations

Most importantly, this would be the first study to evaluate knowledge, attitude, practice, and clinical applications of Regenerative Medicine Therapy among students across public and private institutions in Malaysia. By encompassing students from diverse academic and institutional backgrounds, this study provides a unique, holistic understanding of how different educational settings shape perceptions and awareness of RMT. This comparative perspective is crucial, as it highlights variations in exposure, curriculum emphasis, and accessibility to regenerative medicine education resources between public and private universities and is particularly important given the interdisciplinary nature of the field, which blends biological sciences, technological innovation, engineering principles, and clinical applications. Understanding RMT requires not only foundational scientific literacy but also the ability to think across disciplines, evaluate emerging technologies, and appreciate their clinical and ethical implications. By examining how students from diverse educational contexts engage with these concepts, the study sheds light on the strengths and gaps within Malaysia’s current STEM education.

Another major strength of this research lies in its contribution to the broader framework of STEM education. Regenerative medicine, as an emerging interdisciplinary field, bridges biology, engineering, technology, and clinical sciences, all of which represent the core pillars of STEM education. In these circumstances, regenerative medicine provides an ideal context for assessing the effectiveness of STEM education in nurturing scientifically literate individuals. By identifying the current level of awareness and understanding of regenerative medicine among university students, this research highlights key strengths alongside gaps in existing STEM-based learning approaches. By identifying gaps in awareness and understanding, this underscores the importance of cultivating scientific literacy and critical thinking among young Malaysians to develop the qualities essential to achieving the national agenda of developing a future-ready, innovation-driven workforce.

Additionally, the results of this study have significant implications for the Malaysian Ministry of Education, including strengthening awareness efforts to eradicate misleading information on RMT and developing strategies to achieve profound success in enhancing students’ understanding of RMT application. Beyond formal education, there is an urgent need for nationwide initiatives to enhance public awareness of regenerative medicine and its potential applications. Through these initiatives, policymakers and stakeholders can effectively counteract misinformation, foster informed public discourse, and cultivate a broader understanding of and engagement with RMT among students and the general public.

The study highlights the need for closer collaboration between educational institutions, government bodies, and industry stakeholders in strengthening Malaysia’s STEM and biomedical education ecosystem. As regenerative medicine continues to evolve into a major area of scientific and clinical advancement worldwide, a coordinated and strategic approach is essential to ensure that Malaysia remains competitive in this emerging field. Establishing partnerships between universities and regenerative medicine research centres can facilitate knowledge exchange, internship opportunities, and joint research initiatives, and play a crucial role in nurturing a future workforce. Through joint research projects, students and researchers may contribute to innovation in stem cell science, biomaterials development, tissue engineering, and therapeutic applications.

Policymakers can also leverage the findings of this study to formulate national frameworks that align regenerative medicine education with Malaysia’s long-term scientific and technological development goals. Ultimately, this alignment would not only enhance the quality and relevance of regenerative medicine education but also strengthen Malaysia’s capacity to contribute meaningfully to global scientific progress and biomedical innovation.

However, this study has some limitations. Firstly, the data collected was self-reported by respondents through a questionnaire. Thus, student preferences might change from person to person, and each might have different reasons for choosing, since these underlying reasons are often complex and multifaceted, encompassing personal interests, career aspirations, prior experiences, and socio-cultural influences. Additionally, individual experiences and academic exposure may vary widely among students, especially between those in health-related and non-health-related STEM fields, which could influence their responses and interpretations.

Students in different disciplines may have divergent levels of exposure to specific academic content, research methodologies, or practical experiences, all of which can shape how they perceive and respond to the study. Moreover, as a limitation of the chi-squared test, even trivial relationships can appear statistically significant with a large enough sample. Future studies could improve representation by employing stratified sampling techniques and expanding participation to include students from Peninsular and East Malaysia, thus providing a more comprehensive reflection of the national student population and strengthening the validity of the conclusions.

## Data Availability

The raw data supporting the conclusions of this article will be made available by the authors, without undue reservation.
