# Supplementary material for: Knowledge and attitude of Malaysian university students towards regenerative medicine therapy and their clinical implications: a cross-sectional study
Source: Front Genet. 2026 Jul 28;17:1804348. doi: 10.3389/fgene.2026.1804348 (PMC13456652; doi:10.3389/fgene.2026.1804348)
Supplement: Supplementary file 1 [file Supplementaryfile1.pdf]

INFORMATION SHEET

Project Leader: Dr. Fazlina Nordin, Centre of Tissue Engineering and Tissue Engineering (CTERM), UKM.
Email: nordinf@ppukm.ukm.edu.my

Co-researchers:
Assoc. Prof. Dr. Budi Aslinie Md Sabri, Faculty of Dentistry, UiTM Sungai Buloh Campus.
Assoc. Prof. Dr. Rajesh Ramasamy, Department of Pathology Faculty of Medicine and Health Sciences UPM.
Assoc. Prof. Dr. Deny Susanti, Department of Chemistry, IIUM Kuantan Campus.
Assoc Prof. Dr. Yogeswaran Lokanathan and Dr. Siti Aminah Muhammad Imran, Centre of Tissue Engineering and Tissue Engineering (CTERM), UKM.
Dr. Khairul Bariah Ahmad Amin Noordin, School of Dental Sciences, Health Campus, USM.
Dr. Sharifah Zamiah Syed Abdul Kadir, Department of Pharmacology, UM.

Research Title:
The Knowledge and Attitude of Malaysia Population towards Regenerative Medicine and their clinical implications

Introduction:
You are invited to participate in a research study. Before participating in this study, it is important that you take time to read and understand the information in this Information Sheet.

Purpose of Study:
This survey is hoped to provide the necessary information for government, particularly universities to conduct courses or seminars on regenerative medicine therapy (RMT) to create more awareness on its development and application. Generally, the current study aims to assess the knowledge and attitude of Malaysian population, particularly student attending either public or private universities towards RMT and their therapeutic applications in Malaysia.

Ethical approval: This study has been approved by the UKM ethics committee (Ref: UKM PPI/111/8/JEP-2021-637)

What will the study involve?
This study is a questionnaire-based cross-sectional study. An online questionnaire generated from Google forms was distributed among Malaysian population particularly to students (undergraduates and postgraduates) attending either public or private universities in Malaysia.

Risks and Benefits:
There is unlikely to be a direct risk or/and benefit for you in participating in this study. However, your participation could help us to provide further information for government and non-government sector particularly universities to conduct courses or seminars on RMT to create more awareness on its development and application

Do you have to take part?
Participation in this study is voluntary. If you agree to take part, then you will be asked to sign the “Informed Consent Form” via google form. Should you decide to participate, you can still withdraw from the study without

penalty. Your data will not be used and will be discarded. The researcher may also remove you from the study for a variety of reason. In this event, you will not be penalised or lose your rights as a voluntary.

Data & Confidentiality:
The data from this study will be made into a report which may be published. Access to the data is only by the research team and the REC UKM. The data will be reported in a collective manner with no reference to an individual. Hence your identity will be kept confidential.

Payment and compensation:
You do not have to pay nor will you be paid to participate in this study.

Who can I ask about the study?
If you have any questions, you can direct them to the research team. You can also contact the REC UKM for clarifications.

INFORMED CONSENT FORM

Research Title: The Knowledge and Attitude of Students in Malaysia Public University towards Regenerative Medicine

Researcher's Name: Dr. Fazlina binti Nordin

I, ……………………………………, IC No : ....,

understand that I am being asked to participate in a survey/questionnaire activity for this research. It is my understanding that this questionnaire has been designed to gather information about my knowledge and attitude towards regenerative medicine.

a) have been given time to think about it and all of my questions have been answered to my satisfaction.

b) understand that I may freely choose to withdraw from this study at any time without reason and without repercussion.

c) understand that my anonymity will be ensured in the write-up.

I voluntarily agree to be part of this research study, to follow the study procedures, and to provide necessary information to the doctor, nurses, or other staff members, as requested.

……………………………….   …………………..

(Signature)   (Date)

QUESTIONNAIRE

Understanding Regenerative Therapy

Background

This section is to gather the demographic background of each participant. Please tick (√) at the appropriate box.

1. Age

2. Gender — Male ☐ Female ☐

3. Which state do you currently resides in?

4. Education background — SPM/equivalent ☐ Diploma ☐ Degree ☐ Masters ☐ PhD ☐

5. Field of Study — Architecture ☐ Business ☐ Education ☐ Engineering ☐ Medical and Health Sciences ☐ Law ☐ Others : ____________

6. Are you currently working or studying? — Working ☐ Studying ☐

EDUCATION

7. Which university are you doing your studies? : ______________________

8. Year of study — Year 1 ☐ Year 2 ☐ Year 3 ☐ Year 4 ☐ Year 5 ☐
   Others : ____________

9. What is your course of study — Health/Medical ☐ Non-medical ☐

COURSES/SYLLABUS ASSESSMENT		Strongly Disagree	Disagree	Unsure	Agree	Strongly Agree
10.	The curriculum that I have studied contained good amount of information about Regenerative Medicine Therapy (RMT) and their potential applications in certain diseases and injuries.					
11.	Adding a special course concerning RMT to the curriculum syllabus is advisable.					
12.	I have attended either scientific activities (seminar/forum/courses) or non-scientific activities (public gathering) related to RMT.					
13.	I am interested in attending advanced training course or seminar about RMT if available.					

PREVIOUS EXPERIENCE WITH RMT

14. Have you ever undertake any form of treatment involving regenerative medicine therapy (RMT)? Yes ☐ No ☐

15. If you answered yes, what is the treatment (s) that you have taken before? : ____________________

16. Which among these do you think are RMT? You can choose more than one.

Cord blood banking ☐ Platelet-rich plasma therapy ☐ Bone/skin grafting ☐ Fruit/vegetables stem cells ☐ Anti-aging therapy ☐ Bone marrow transplant ☐

Chimeric Antigen Receptor T-cell (CAR-T) therapy ☐ Allogenic mesenchymal stem cell therapy ☐

Knowledge and Attitude towards Regenerative Medicine Therapy (RMT)

This section aims to assess your level of knowledge and understanding on RMT, as well as your attitude towards its practices. Please rate your level of agreement with the following statements by checking (√) appropriate box:

BASIC KNOWLEDGE		True	False	I don't know
17.	RMT involves three (3) main niches of research which are i) Stem Cell Therapy, ii) Tissue Engineering, and iii) Biomaterials.			

18.	Which are the niche of RMT mentioned below is the most prominent or common to your knowledge? (Rate from 1-3)	**1**	**2**	**3**
	**Stem cell therapy**			
	**Tissue Engineering**			
	**Biomaterials**			

BASIC KNOWLEDGE		True	False	I don't know
19.	***Stem Cell Therapy*** is the use of stem cells to treat or prevent a disease or condition.			
20.	***Tissue engineering*** uses cells, materials and engineering to treat diseases.			
21.	A ***biomaterial*** is a substance that has been engineered to interact with biological systems for a medical purpose.			
22.	RMT is a treatment of certain diseases (such as cancer related disease) and injuries (cells/tissues/organs related injuries and replacement).			
23.	There is no specific legislation on the conservation and use of stem cells in RMT?			

ATTITUDE ASSESSMENT		Strongly Disagree	Disagree	Unsure	Agree	Strongly Agree
24.	Do you think that you have enough knowledge on RMT research and their potential applications in treatment of certain diseases and injuries?					
25.	There are good amount of information about RMT and their potential applications in certain diseases and injuries made available to public.					
26.	Do you agree that RMT research should be carried out in Malaysia?					
27.	Do you think that RMT research has a positive future?					
28.	The use of treatment related RMT in medical treatments contradicts with human ethics and religious principle.					
29.	I will recommend treatment related to RMT if it is available to my family/friends/public.					
30.	I am aware of RMT research and its potential applications in treating certain diseases and injuries.					
31.	There should be more public awareness programs about RMT and their therapeutic application should be made available.					
32.	Do you think that the government should put more funding in research related to RMT?					
33.	Some people believe that life begins at conception, and that to destroy a human embryo would be immoral, even to save or reduce suffering in a existing human life. Do you agree with this statement?					
34.	Many religions also oppose stem cell research and treatment. Do you think it's acceptable for some parents to refuse stem cell treatment for their children as it goes against their religious beliefs, even if the treatment could be life-saving?					
35.	Would you be interested in developing your knowledge about stem cells?					

BASIC UNDERSTANDING ON STEM CELLS		True	False	I don't know
36.	Stem cells are unspecialised type of cells which are capable of forming any cell type.			
37.	Human sperms and eggs are considered a source of adult stem cells.			
38.	Stem cells obtained from adults are specialised cells that can form either bone or cartilage only.			
39.	Embryonic stem cells can be obtained from umbilical cord.			
40.	Stem cells obtained either from tissues or organs (such as liver, pancreas, skin, dental, etc) are considered as adult stem cells.			
41.	Adult bone marrow stem cells are usually taken from the spine.			
42.	Autologous transplant of adult stem cells can fail mainly because of immunogenic reaction.			
43.	Chondrocytes are considered as one type of stem cells.			
44.	Dental implants derived from stem cells are now available to replace missing teeth.			
45.	Stem cell banks are now available in Malaysia.			
46.	Three (3) types of stem cells are i) adult stem cells, ii) embryonic (or pluripotent) stem cell, and iii) induced pluripotent stem cell (iPSC).			
47.	The embryonic stem cells are capable to differentiate into the three (3) germs layers **i) ectoderm, ii) mesoderm, and iii) endoderm**.			

This is the end of the questionnaire. Thank you for your participation in our study.

Supplementary 2

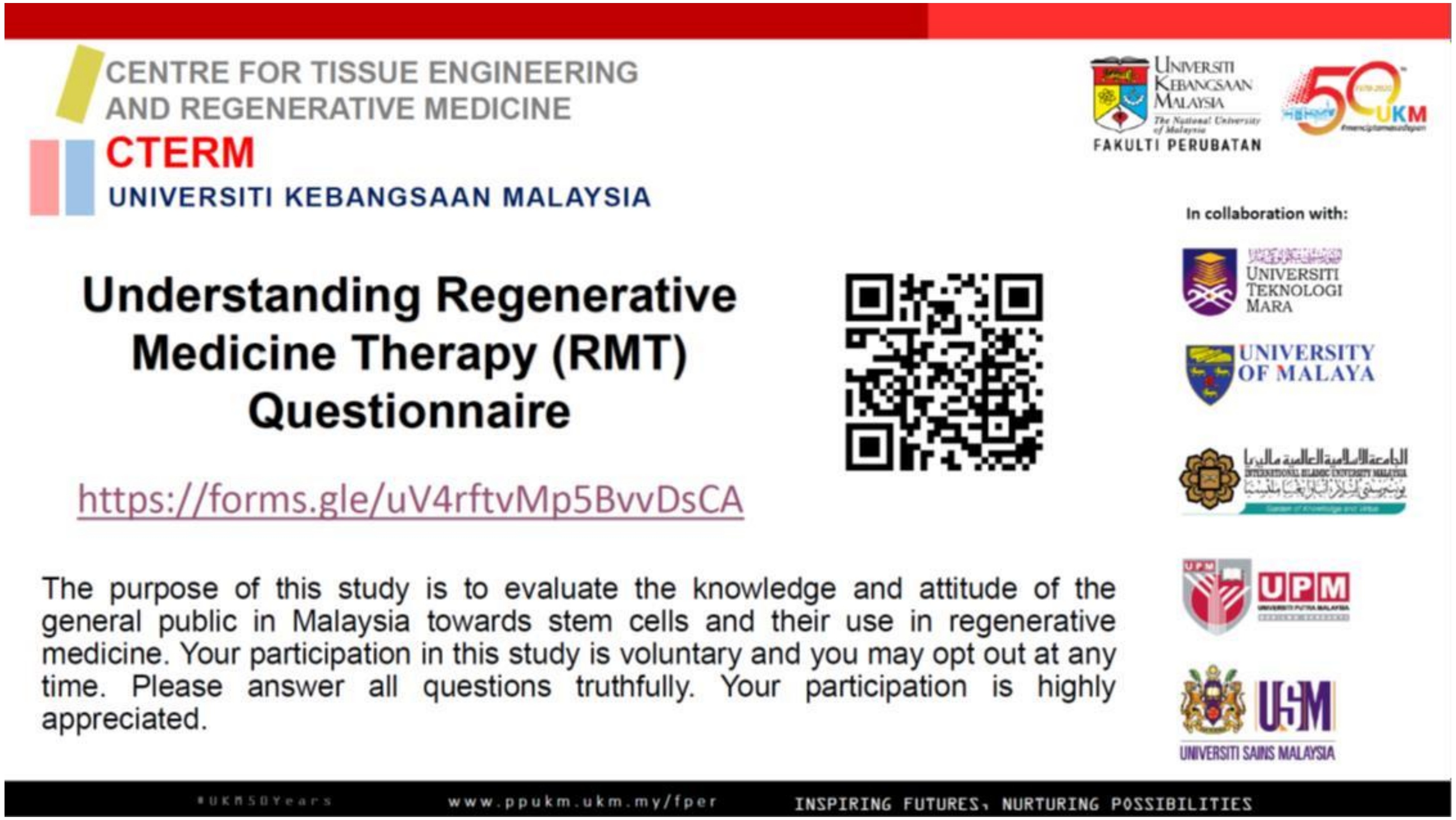

Supplementary [redacted] 3

Items	Relevant (Rating 3 and 4)	Not relevant (Rating 1 and 2)	I-CVIs	Interpretation
**Background**				
Age	6	0	1	Appropriate
Gender	6	0	1	Appropriate
Education Background	6	0	1	Appropriate
Field of Study	6	0	1	Appropriate
Are you currently working or studying	6	0	1	Appropriate
**Education**				
Which university are you doing your studies	5	1	0.928	Appropriate
Year of study	5	1	0.928	Appropriate
What is your course ofstudy	6	0	1	Appropriate
**Course/Syllabus Assessment**				
1	6	0	1	Appropriate
2	6	0	1	Appropriate
3	6	0	1	Appropriate
4	6	0	1	Appropriate
**Previous experience with RMT**				
1	6	0	1	Appropriate
2	6	0	1	Appropriate
3	6	0	1	Appropriate
**Knowledge and Attitude**				
Basic knowledge				
1	6	0	1	Appropriate
2	6	0	1	Appropriate
3	6	0	1	Appropriate

4	6	0	1	Appropriate
5	6	0	1	Appropriate
6	6	0	1	Appropriate
7	6	0	1	Appropriate
Attitude Assessment				
1	6	0	1	Appropriate
2	6	0	1	Appropriate
3	6	0	1	Appropriate
4	6	0	1	Appropriate
5	6	0	1	Appropriate
6	6	0	1	Appropriate
7	6	0	1	Appropriate
8	6	0	1	Appropriate
9	6	0	1	Appropriate
10	6	0	1	Appropriate
11	6	0	1	Appropriate
12	6	0	1	Appropriate
**Basic Understanding on Stem Cell**				
1	6	0	1	Appropriate
2	6	0	1	Appropriate
3	6	0	1	Appropriate
4	6	0	1	Appropriate
5	6	0	1	Appropriate
6	6	0	1	Appropriate
7	6	0	1	Appropriate
8	6	0	1	Appropriate
9	6	0	1	Appropriate
10	6	0	1	Appropriate
11	6	0	1	Appropriate
12	6	0	1	Appropriate

Table 1 Calculation of CVIs to validate the questionnaire. Number of items considered relevantby all the panelists=44, Number of items=46, Average of CVIs=0.997, Number of experts=6
